# MeioSeed: a CellProfiler-based program to count fluorescent seeds for crossover frequency analysis in *Arabidopsis thaliana*

**DOI:** 10.1186/s13007-018-0298-3

**Published:** 2018-04-18

**Authors:** Niels van Tol, Martijn Rolloos, Peter van Loon, Bert J. van der Zaal

**Affiliations:** 1 Faculty of Science, Institute of Biology Leiden, Leiden University, Sylviusweg 72, 2333 BE Leiden, The Netherlands; 2 Rijk Zwaan Breeding BV, Eerste Kruisweg, 4793 RS Fijnaart, The Netherlands

**Keywords:** *Arabidopsis*, Meiosis, Crossover frequency, Fluorescent seed reporter, Seed counting, Program, Salt stress

## Abstract

**Background:**

The formation of crossovers during meiosis is pivotal for the redistribution of traits among the progeny of sexually reproducing organisms. In plants the molecular mechanisms underlying the formation of crossovers have been well established, but relatively little is known about the factors that determine the exact location and the frequency of crossover events in the genome. In the model plant species *Arabidopsis*, research on these factors has been greatly facilitated by reporter lines containing linked fluorescence marker genes under control of promoters active in seeds or pollen, allowing for the visualization of crossover events by fluorescence microscopy. However, the usefulness of these reporter lines to screen for novel modulators of crossover frequency in a high throughput manner relies on the availability of programs that can accurately count fluorescent seeds. Such a program was previously not available in scientific literature.

**Results:**

Here we present MeioSeed, a novel CellProfiler-based program that accurately counts GFP and RFP fluorescent *Arabidopsis* seeds with adjustable detection thresholds for fluorescence intensity, making use of a robust seed classifier which was trained by machine learning in Ilastik. Using the previously published reporter line Col3-4/20 as an example, we explain the use of MeioSeed and the steps taken to optimize the thresholding settings of the program to fit the published model for recombination frequency and transgene segregation. The use of MeioSeed is illustrated by investigating salt stress as a novel abiotic trigger for changes in crossover frequency in Col3-4/20 (♂) × Ler-0 (♀) F_1_ hybrids. Salt stress was found to trigger increases in crossover frequency between the marker genes of up to 70% compared to the control treatment without salt stress. Genotyping of control and salt treated populations revealed that the changes in crossover frequency were not limited to the region between the marker genes, but that fluctuations in crossover frequency are likely to occur genome-wide after treatment with high salt concentrations.

**Conclusions:**

MeioSeed allows for the high throughput recognition and counting of fluorescent *Arabidopsis* seeds and can facilitate the screening for novel abiotic and biotic modulators of crossover frequency using reporter lines in *Arabidopsis*.

**Electronic supplementary material:**

The online version of this article (10.1186/s13007-018-0298-3) contains supplementary material, which is available to authorized users.

## Background

For sexually reproducing eukaryotic organisms the process of meiosis is pivotal for the formation of haploid gamete cells and for the redistribution of the genetic material among the progeny. Meiosis consists of two consecutive cell divisions [1], with the first one (meiosis I) resulting in the separation of homologous chromosomes usually accompanied by meiotic crossing-over events between non-sister chromatids. During the second division (meiosis II) the sister chromatids are separated, resulting in four haploid daughter cells each with a unique reassembly of the genetic properties of the diploid progenitor meiocyte.

An essential step in the accurate redistribution of chromosomes during meiosis I is the correct formation of pairs of homologous chromosomes (bivalents), which is mechanically facilitated by the invasion of strands of homologous chromosomal DNA at double stranded break sites generated by the transesterase SPO11 [2, 3], resulting in 5′ end resection of the breaks and the formation of D-loops [1, 4]. The resolution of D-loops can result in the reciprocal exchange of large parts of the chromosome arms, events which are called crossovers [1, 4]. Crossovers are of special interest to plant breeders, as they allow for the redistribution of allelic variants among populations of F_2_ plants. The pathways for the resolution of D-loops into crossovers or non-crossovers have been well established [1, 4, 5], but relatively little is known about the factors that determine which SPO11 breaks are resolved into crossovers and how the frequency of crossovers along the genome is determined.

Studies on crossover frequency in plants make use of different types of approaches to visualize crossover events. Fundamental biological questions on the formation of bivalents and crossovers are often addressed by cytogenetic studies to count chiasmata or foci for markers of double stand break formation with microscopy [6–10]. The main advantage of these cytogenetic techniques is that the formation of crossovers can be studied in vivo, but they require extensive expertise [11–13]. Perhaps the most widely used approach is PCR-based genotyping (e.g. Kompetitive Allele Specific (KASP) genotyping [14]) of hybrid F_2_ plants with primer sets for genetic markers containing single nucleotide polymorphisms (SNPs) among the parental accessions. When genotyping is done for multiple different markers along the chromosome and for a large number of F_2_ individuals, a genetic map can be constructed which gives an overview of the crossover landscape of the genome. The main advantage of the genotyping approach is that primers can be designed for any polymorphic locus in the genome and that there are ample databases with established SNP markers that have been made publicly available (e.g. [15]). However, the high throughput application of genotyping requires large numbers of DNA extractions and PCR reactions and all the required equipment. Finally, the research on crossover frequency in the model species *Arabidopsis* has been greatly facilitated by the availability of fluorescence reporter lines for all five chromosomes [16–18]. These lines contain *in cis* linked fluorescence reporter constructs under control of seed or pollen specific promoters and are separated by known genetic distances, thereby enabling the visualization of crossover events between the two fluorescence marker constructs in the F_2_ seeds or in the pollen of F_1_ plants, respectively. The main advantage of this approach is that it in principle only relies on the availability of a fluorescence microscope.

**Fig. 1:**
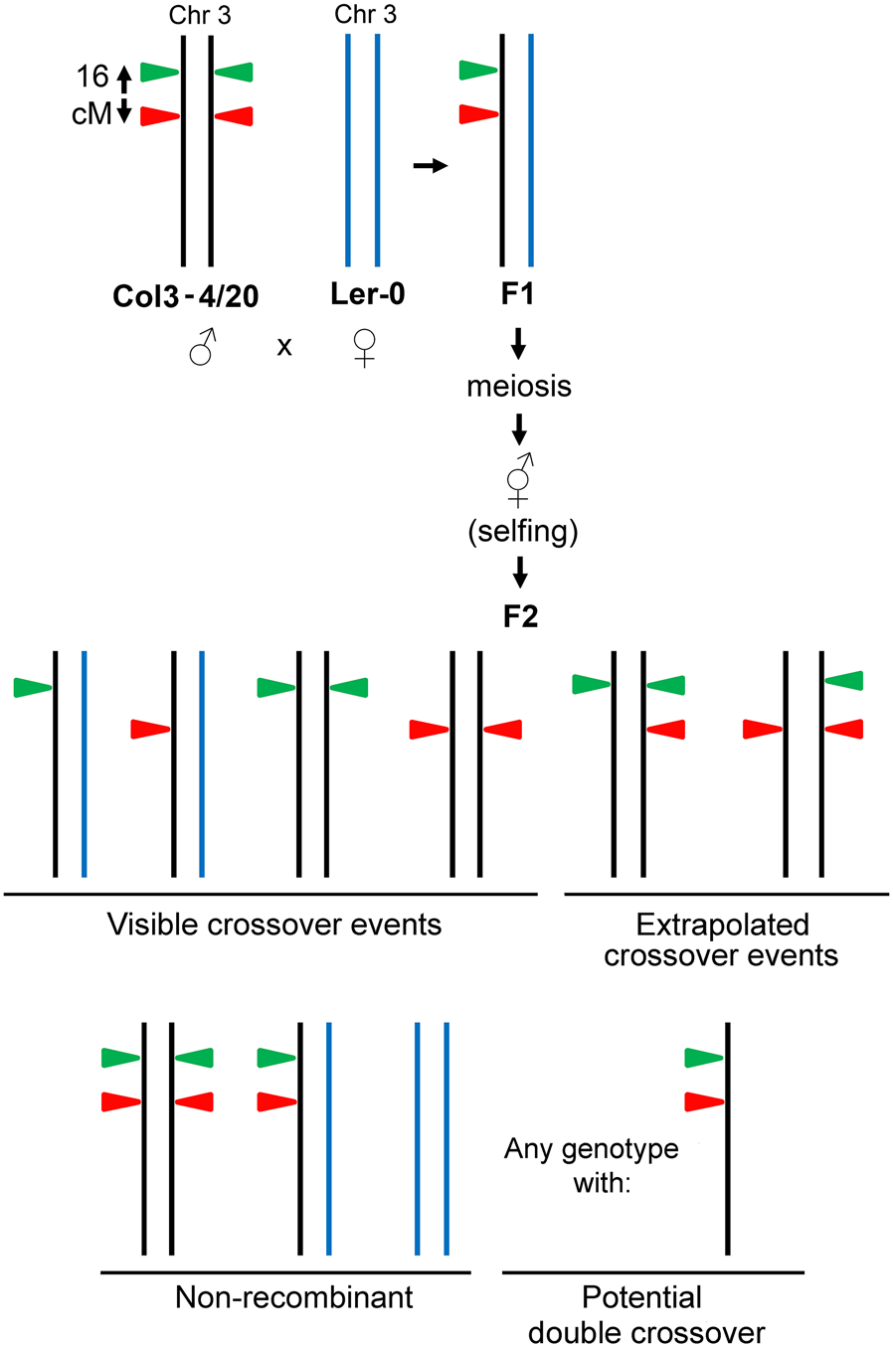
Overview of the Col3-4/20 (♂) × Ler-0 (♀) crossing scheme and possible outcomes of meiotic recombination events and subsequent chromosome segregation. Meiotic reporter line Col3-4/20 (♂) harboring *RFP* and *GFP* reporter constructs at the top of the long arm of chromosome 3 (separated by a genetic distance of 16 cM) is crossed with the accession Ler-0 (♀). The resulting F_1_ hybrids are selfed and their F_2_ seeds are collected. Overall crossover frequencies are calculated from RFP and GFP fluorescence images using the formula for crossover frequency described previously [16]. Crossover events referred to as ‘visible crossover events’ can be observed directly as the F_2_ seeds displaying either only RFP or only GFP signal. Crossover events referred to as ‘extrapolated crossover events’ are not directly visible based on the fluorescence images only. The frequency of these events is extrapolated from the fluorescence images based on theoretical allele frequencies as described previously [16]. For the sake of clarity of the figure the chromosomes are presented in the colors of the original parent throughout the crossing scheme (black for Col3-4/20 and blue for Ler-0)

The meiotic reporter line Col3-4/20 [16] is often used in studies related to crossover frequency in *Arabidopsis* [19–23], and contains *in cis* linked *RFP* and *GFP* reporter constructs at the top of the long arm of chromosome 3, separated by a genetic distance of ~ 16 cM (Fig. 1). The expression of these constructs is driven by the seed-specific napA promoter, allowing for visualization of crossover events occurring between the marker genes in the F_2_ seed pool with fluorescence microscopy [16]. When the homozygous Col3-4/20 reporter line is crossed with another homozygous accession (e.g. Ler-0; Fig. 1) and the F_1_ plants are subsequently selfed, the frequency of recombinant and non-recombinant chromosomes among the F_2_ seeds (Fig. 1) can be calculated as described previously [16]. Furthermore, the frequency of recombinant chromosomes of which detection is masked by non-recombinant chromosomes harboring both marker genes or by double recombinant chromosomes (Fig. 1) can be extrapolated based on theoretical allele frequencies and transgene segregation patterns [16]. With the fraction of recombinant single-colored seeds as cue, the genetic distance in centimorgan (cM) can subseqently be calculated using a mapping function of choice. Reporter lines such as Col3-4/20 are therefore useful tools to study the effects of biotic and abiotic treatments on crossover frequency in *Arabidopsis*. Their use would be greatly facilitated by the availability of high throughput seed counting algorithms with accurate recognition of seeds and adjustable detection limits for fluorescence. Such an algorithm was previously not available in scientific literature.

Here, we present a novel, high throughput program to count fluorescent *Arabidopsis* seeds, which we have called MeioSeed. The program consists of a batch-wise image processing algorithm in the freeware program CellProfiler, combined with a seed classifier trained by machine learning in the program Ilastik. Using the meiotic reporter line Col3-4/20 as an example, we show that MeioSeed accurately counts red and green fluorescent seeds with adjustable detection thresholds for fluorescence from microscopy images, giving overlay images with seed counts and fluorescence colors and a raw CSV data file as an output. We further illustrate how we optimized the pipeline for *Arabidopsis* seeds in our setup, which could be adjusted for any other fluorescence microscopy setup. Finally, we illustrate the use of MeioSeed by investigating salt stress as a novel abiotic trigger for changes in crossover frequency in Col3-4/20 (♂) × Ler-0 (♀) F_1_ hybrids.

## Methods

### Plant material and growth conditions

Homozygous seeds of the meiotic tester line ‘Col3-4/20’ [16] harboring *in cis* linked napA::*GFP* (insertion site: 256,516 bp; 4 cM) and napA::*RFP* (insertion site: 5,361,637 bp; 20 cM) fluorescence reporter constructs at the top of the long arm of chromosome 3 [16] were kindly provided by Dr. Cathy Melamed-Bessudo and Prof. Avraham A. Levy (Department of Plant Sciences, Weizmann Institute of Science, Rehovot, Israel). Col3-4/20 (♂) plants were crossed with the accession Landsberg *erecta* (Ler-0; ♀) to obtain F_1_ hybrids, which were selfed to obtain pools of F_2_ seeds for measurements of crossover frequency and/or genetic mapping. F_1_ hybrids of which data are presented in Fig. 3 were grown in a climate-controlled growth chamber at 20 °C, 70% relative humidity, a 16 h photoperiod and at ~ 150 μmol m^−2^ s^−1^ of photosynthetically active radiation (PAR). The F_1_ hybrids which received NaCl treatments and control treatments with 0 mM NaCl (data presented in Fig. 4) were grown in a climate-controlled growth chamber at 20 °C, 70% relative humidity, a 12 h photoperiod and at ~ 200 μmol m^−2^ s^−1^ PAR. Rapeseeds (*Brassica napus* cv. Westar) were kindly provided by Arezoo Rahimi (Department of Molecular and Developmental Genetics, Leiden Institute of Biology, Leiden, The Netherlands). Barley grains (*Hordeum vulgare*) were kindly provided by Fytagoras B.V. (Leiden, The Netherlands).

### Collection of seed images

Bright field images, and GFP and RFP fluorescence images of *Arabidopsis* seeds were collected using a LeicaMZ16FA fluorescence stereomicroscope **(**Leica, Wetzlar, Germany). F_2_ seeds were placed on the microscope platform by uniformly distributing them on a sheet of paper in a line-shape with a diameter of ~ 1.5–2 cm (in total approximately 3000–5000 seeds). By moving the platform of the microscope following this line at a zoom drive of 7.17×, ten non-overlapping bright field (exposure: 1.1 s, gain: 1.0, saturation: 1.0, gamma: 1.21), RFP (exposure: 1.1 s, gain: 1.0, saturation: 1.0, gamma: 1.21) and GFP (exposure: 4.0 s, gain: 1.0, saturation: 1.0, gamma: 1.21) images were captured and stored as 2592 × 1944 pixel^2^ TIFF files with the 1.5 × scaling setting applied. The automatic white balance function was applied on the bright field images and switched off during capturing of the fluorescence images. The same settings were used for all images collected. Bright field images of rapeseeds and barley grains were collected using a fixed digital camera setup (Canon EOS 1100D). Because fluorescence images could not be captured for these species, the bright field images were also used as input for fluorescence classification to allow normal operation of MeioSeed.

### Construction of the MeioSeed package and data analysis

The MeioSeed image analysis workflow was based on the combined [24] use of a CellProfiler (version 2.1.1; [25]) pipeline with an integrated Ilastik (version 0.5.12; [26]) classifier, enabling recognition and segmentation of seeds by machine learning. A random forest classifier was used in the interactive machine learning step based on user-drawn image labels on ‘White’ fluorescence stereomicroscopy images. After training the classifier was exported for integration in the CellProfiler pipeline. Further details on the operation of MeioSeed are provided in the results section. The MeioSeed package is available at http://cellprofiler.org/examples/published_pipelines. Smoothly running MeioSeed requires at least a quadcore processor. The TIFF microscopy images used as input for MeioSeed should have a format of 2592 × 1944 pixel^2^.

### Optimization of the MeioSeed thresholding settings

Optimization of the MeioSeed ‘Filter objects’ thresholding settings was performed using fluorescence stereomicroscopy images of F_2_ seeds from ten independent Col3-4/20 (♂) × Ler-0 (♀) F_1_ hybrids. This data set was run in the MeioSeed program on a trial-and-error basis with different ‘FilterObjects’ settings for the detection of red and green fluorescence until the output of the program gave the best possible fit with the model for crossover frequency described previously [16]. Some of the different combinations of settings which were tried are presented in Fig. 3. In order for the best fit to be achieved, a filtering step was introduced to remove microscopy images which had a clear bias for RedOnly seeds compared to GreenOnly, or vice versa, as theoretically there is no reason to assume that the RedOnly:GreenOnly ratio should be different from 1:1. To this end, images with a higher than fourfold skewed RedOnly to GreenOnly ratio (or vice versa) were disregarded for the data analysis in subsequent experiments described below. This threshold was chosen arbitrarily.

### NaCl treatments

At 42 ± 2 days after germination all open flowers, open flower buds and siliques were removed from Col3-4/20x Ler-0 F_1_ hybrids to ensure that only meiotic crossover events after the NaCl treatments were represented among the F_2_ seeds. Subsequently, the plants were watered from below with fixed volumes of NaCl solutions of the indicated concentrations in demineralized water. This treatment was done once. Photos of the plants were taken every 4 days to document the effect of salt stress on the developing primary inflorescences. After seed set, the complete F_2_ seed pool of the primary inflorescence of each treated plant was harvested and subjected to fluorescence stereomicroscopy as described above. The distribution of the raw crossover frequency data of the 0 mM NaCl control treatments was visually examined with histograms and Q–Q plots, and was concluded to be normal. The data from the other treatments were then normalized to the control treatment with 0 mM NaCl to combine data sets, and statistically analyzed with the heteroscedastic *T* test function of Microsoft Excel assuming unequal variance between samples. A *p* value of 0.05 was used as a threshold for significance.

### KASP genotyping

KASP genotyping was performed as described previously [27] on genomic DNA extracted from a single leaf (diameter of ~ 1 cm) of 192 F_2_ plants for 0 mM NaCl and 300 mM NaCl treatments, respectively (therefore n = 384 chromosomes per treatment), using the same set of SNP probes. Genetic maps were constructed using the program JoinMap [28].

## Results

### Composition and operation of MeioSeed

**Fig. 2:**
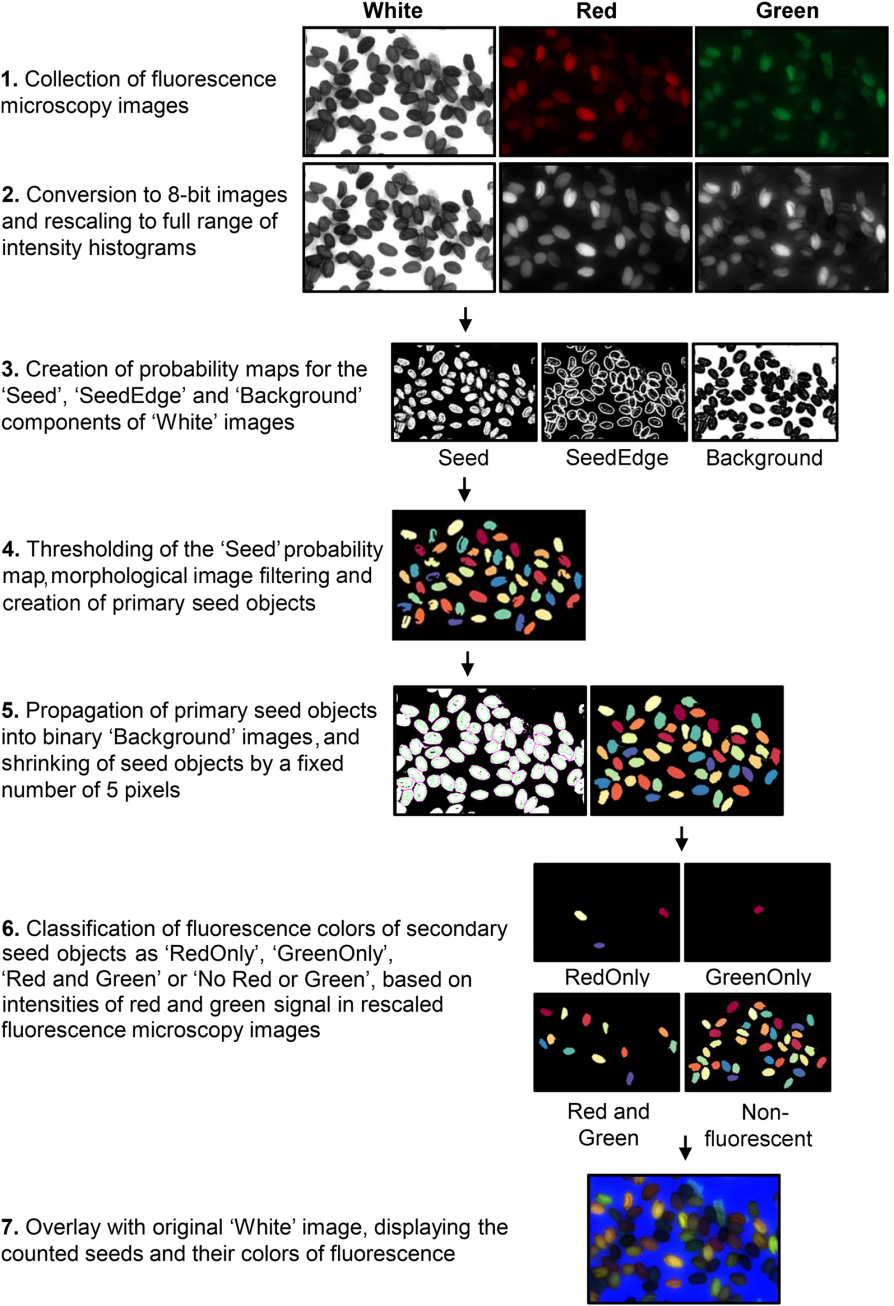
Overview of the different steps in the MeioSeed algoritm to convert fluorescence stereomicroscopy images into overlay images with counted seeds and assigned fluorescence colors

A batch-wise image processing pipeline was created in the program CellProfiler, making use of a machine-learning based classifier to distinguish individual seeds in microscope images and to separate them from the background. The classifier was created with the program Ilastik, making use of ‘White’ stereomicroscopy images of F_2_ seeds (Fig. 2, step 1) to define three classes of image components: the seed (‘Seed’), the edge of the seed (‘SeedEdge’), or the background (‘Background’). The classifier takes intensity, texture and edge features of seeds as input for the random-forest based pixel classification algorithm. In an iterative process of labelling, training and adding badly classified parts of microscopy images to the training set, a robust classifier was created. The classifier was then exported into CellProfiler as a HDF5 file. An overview of the different steps in the MeioSeed algorithm is provided in Fig. 2. More details on the operation of MeioSeed and the algorithm are provided in Additional file 1. MeioSeed generates .tiff images in the designated output folder in which the seed counts and fluorescence signals are displayed as overlays on the original ‘White’ microscopy images (Fig. 2, step 7). A quantification of the counting is given as a CSV file in the output folder ‘Data’, which can be imported into for instance Microsoft Excel for data analysis.

### Optimization of ‘FilterObjects’ thresholding settings for red and green fluorescence

**Fig. 3:**
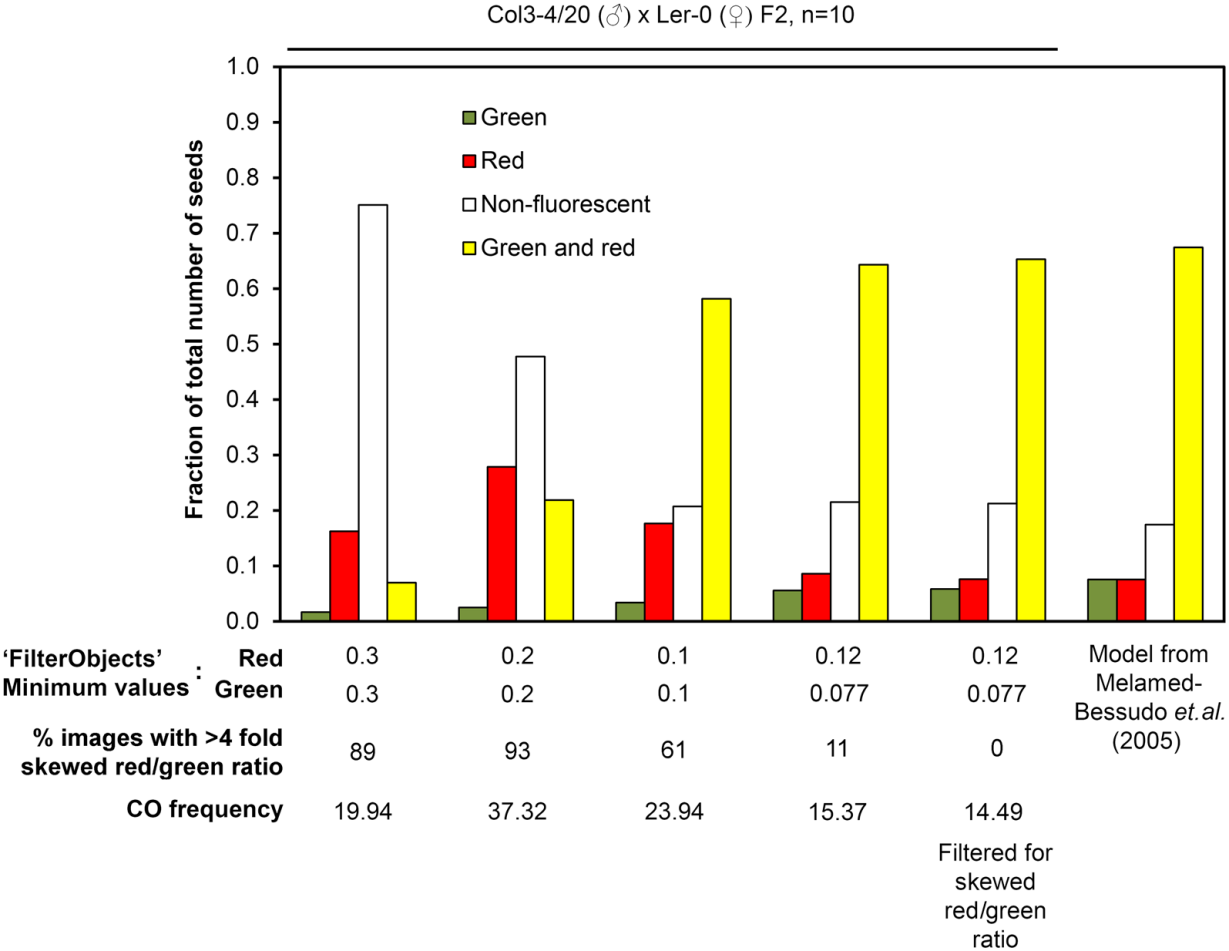
Optimization of the MeioSeed thresholding settings to acquire a fit with the model for crossover frequency described by Melamed-Bessudo et al. [16]. A set of 100 brightfield, RFP and GFP fluorescence stereomicroscopy images collected from ten independent Col3-4/20 (♂) × Ler-0 (♀) F_2_ populations were run in MeioSeed with different combinations of ‘FilterObjects’ thresholding settings for RFP and GFP fluorescence. Presented are the counted fractions of which are green, red, green and red or non-fluorescent seeds among the total number of seeds, along with the calculated crossover frequencies. The theoretical fractions according to the model are also provided. The best fit with the model was achieved with the ‘FilterObjects’ settings of 0.12 for red fluorescence, 0.077 for green fluorescence and subsequent removal of photos with a ≥ fourfold scewed ratio of red:green fluorescent seeds (or vice versa)

For the accurate detection of seeds with recombinant chromosomes (red or green fluorescent only) it is important that the fluorescence detection thresholds are well chosen, so that false positive seeds (e.g. due to autofluorescence) are disregarded for the data analysis. In our experience, Col3-4/20 seeds and Col3/4-20 × Ler-0 hybrid seeds are not autofluorescent in the RFP channel and only slightly so in the GFP channel, but this could be different for other reporter lines. In addition, it is important that the occurrence of red and/or green fluorescence among the seeds is in accordance with the theoretical segregation pattern of the marker genes. In order for this to be achieved we optimized the ‘FilterObjects’ settings for red and green fluorescence to match the theoretical fractions of the seed population as previously described [16]. On a trial-and-error basis, different combinations of ‘FilterObjects’ settings for red and green fluorescence were tested on ten independent populations of F_2_ seeds resulting from crosses between Col3-4/20 (♂) and Ler-0 (♀). An overview of the resulting fits with the model is provided in Fig. 3, along with the calculated crossover frequencies based on the seed counts. The best fit could be achieved with a ‘FilterObjects’ threshold of 0.12 for red and 0.077 for green fluorescence, which resulted in a crossover frequency of 15.39% (Fig. 3) compared to the 16% described previously [16]. However, we observed that in some microscopy images there was a clear discrepancy between the number of RedOnly and GreenOnly seeds, whereas the ratio between these numbers is theoretically expected to be 1:1. To create a more reliable fit with the model we therefore opted to introduce a filtering step in Microsoft Excel to disregard images with a fourfold or higher skewed RedOnly:GreenOnly ratio, or vice versa. This threshold was chosen arbitrarily, and resulted in a slightly improved fit with the model (Fig. 3). The ‘FilterObjects’ settings of 0.12 for red fluorescence, 0.077 for green fluorescence and the subsequent filtering step were used for the data analysis described below, but are likely to be different when other microscope settings are used.

### Example: using MeioSeed to investigate salt stress as a novel trigger for increases in crossover frequency in *Arabidopsis*

To illustrate the use of MeioSeed we investigated whether it can be used to accurately detect changes in crossover frequency and whether it can be used to find novel treatments to modulate crossover frequency in *Arabidopsis*. To this end, we investigated whether salt stress as a known abiotic trigger for large scale chromatin remodeling [29, 30] and anther development [31] of *Arabidopsis* could be used as a novel abiotic trigger of changes in crossover frequency.

**Fig. 4:**
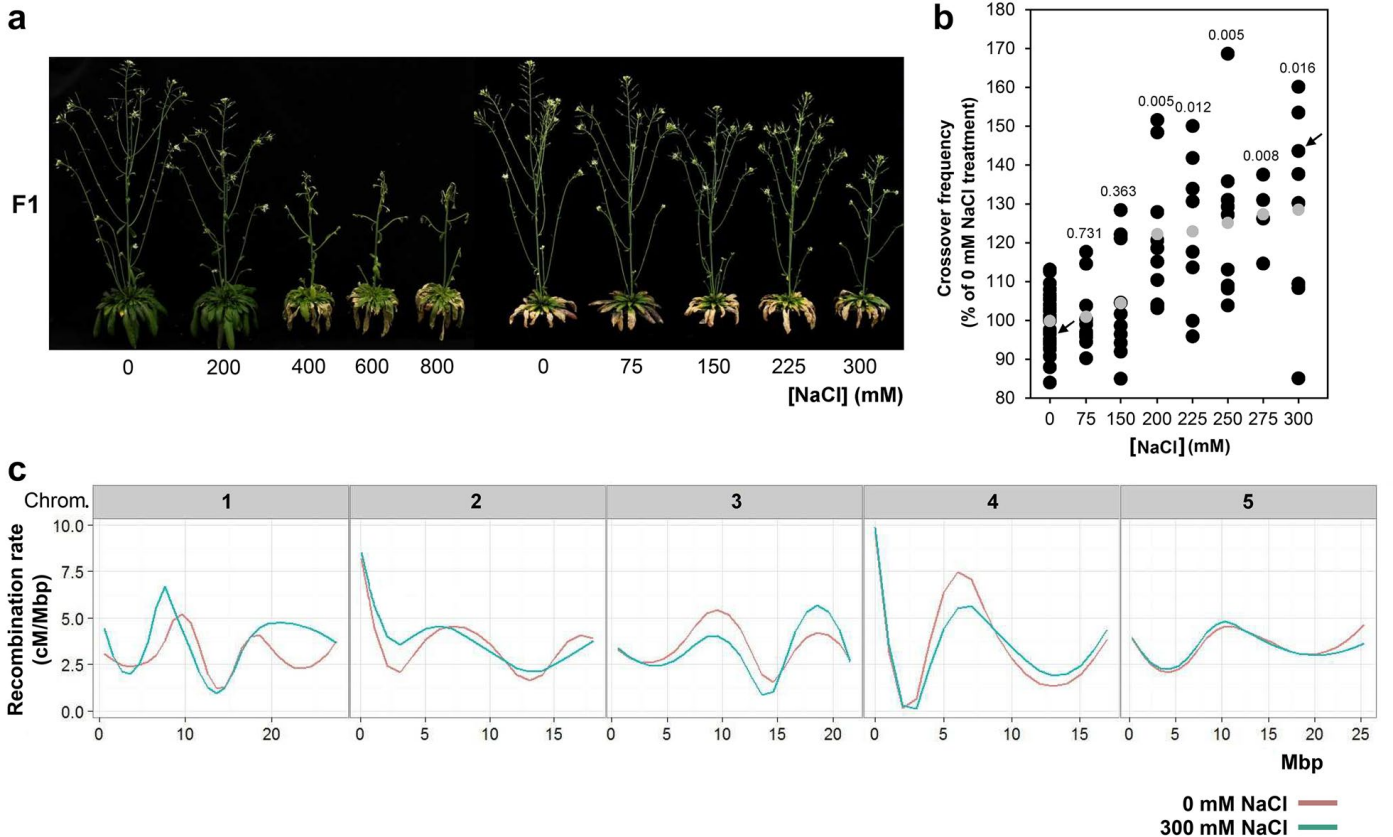
Analysis of the effect of systemic salt stress on the meiotic crossover frequency of F_1_ Col3-4/20 (♂) × Ler-0 (♀) hybrids. **a** Photos of F_1_ hybrids 8 days after treatments (50 days post germination) with NaCl solutions of the indicated concentrations in demineralized water. **b** Frequency of meiotic crossovers between the GFP and RFP fluorescence markers at the top of chromosome 3 detected in the F_2_ seeds. Black dots represent the crossover frequencies of individual F_1_ hybrids (n ≥ 8 per treatment, except for 275 mM NaCl in which case n = 4). Crossover frequencies were normalized to the control treatment with 0 mM NaCl to combine data sets. Grey dots represent the average crossover frequency. F_1_ hybrids of which F_2_ KASP genotyping data are presented in panel C are indicated by black arrows. **c** Genome-wide KASP genotyping analysis on the F_2_ seedling progeny of F_1_ hybrids treated with either 0 mM NaCl or 300 mM NaCl. Plotted is the recombination rate per Mbp on the physical map based on 39 SNP marker sets

Firstly, Col3-4/20 (♂) × Ler-0 (♀) F_1_ hybrids were watered at 42 days post germination with NaCl solutions ranging from 0 mM to 800 mM to determine the NaCl treatment which would allow for the highest level of salt stress while still allowing the plants to set seeds. From these experiments we noted that 300 mM NaCl was the highest concentration at which viable seeds could still be set (Fig. 4a). While fertility of F_1_ hybrids treated with 300 mM NaCl in terms of total seed load was on average 57% higher than for hybrids treated with 0 mM NaCl, the germination percentage of the F_2_ seeds was much lower (approximately 25% versus 100% after treatment with 0 mM NaCl), indicating that the ions also accumulate in the seeds. Subsequently, the F_2_ seeds of hybrids treated with concentrations of NaCl between 0 and 300 mM (Fig. 4a) were harvested and fluorescence stereomicroscopy images were collected (≥ 1000 seeds per F_1_ hybrid). The images were then analyzed with MeioSeed to calculate the crossover frequency per F_2_ seed pool, the results of which are presented in Fig. 4b. We observed that the variation in crossover frequency significantly increased after treatments with NaCl concentrations of 200 mM and higher (Fig. 4b), and that there was on average a positive correlation between the NaCl concentration and crossover frequency, in some cases allowing for increases in crossover frequency of up to 70% of the control treatment with 0 mM NaCl. These data demonstrated that salt stress can affect crossover frequency in *Arabidopsis* between the two fluorescent markers in the Col3-4/20 reporter line at the top of the long arm of chromosome 3. To examine whether salt stress also triggered variation in crossover frequency at other loci in the genome, we performed KASP genotyping with a set of 39 previously described SNP markers polymorphic for Col-0 and Ler-0 [27] to construct genetic maps of two F_2_ populations resulting from 0 mM NaCl and 300 mM NaCl treatment, respectively (provided in Additional file 2). When these data were plotted against the physical map of the genome, we noted that salt treatment indeed led to genome-wide fluctuations in crossover frequency (Fig. 4c).

Altogether these data showed that crossover analysis of seed fluorescence reporter lines with MeioSeed could be used to screen for novel treatments or other approaches to modulate crossover frequency between reporter loci, but also at other loci in the genome.

### Using MeioSeed for counting other seed species

The focus of this study was on *Arabidopsis* seeds because fluorescent seed reporter lines with *in cis* linked reporter constructs are at present exclusively available for *Arabidopsis*. Should fluorescent seed reporter lines become available for other model plant species or crop species as well, we were interested whether MeioSeed could also be used to count seeds of other plant species. To this end, we generated seed classifier files for rapeseeds (*Brassica napus* cv. Westar), which are similar to *Arabidopsis* seeds in shape, but larger and rather heterogeneous in size, and for barley grains, which are much larger and have a more complex morphology, which might be more difficult to recognize consistently. We subsequently captured bright field images of these seeds using a digital camera setup, because stereomicroscopy is not feasible for capturing images of larger seeds, and used these images as an input for seed counting in MeioSeed. As fluorescence images could not be collected for these species, the bright field images were run in triplicate as if they were fluorescence images, to allow for normal operation of MeioSeed as for *Arabidopsis* seeds. The classifier which was generated for rapeseeds was very robust and allowed for efficient counting of a heterogeneous pool of rapeseeds while excluding flower debris from the analysis (Additional file 3), while otherwise using the same settings as for *Arabidopsis* seeds. The classifier generated for barley grains was less robust (Additional file 4), likely due to the more complex morphology of the grains and the ridges in the seed coat, which hampered recognition. Regardless, a significant fraction of the barley grains could be counted. Together, these data showed that MeioSeed is in principle compatible with other types of seeds and images, provided that a classifier file is trained by machine learning in Ilastik and that the morphology of the seeds is not too complex.

## Discussion

Here, we have presented MeioSeed, a novel high throughput program to count *Arabidopsis* seeds from fluorescence microscopy images. Using the meiotic reporter line Col3-4/20, we showed that MeioSeed accurately counts red and green fluorescent seeds using an Ilastik-based seed classifier with adjustable detection thresholds for fluorescence. Furthermore, we demonstrated the optimization of the fluorescence threshold settings to fit the known model for recombination frequency and transgene segregation in the F_2_ seed progeny of crosses with Col3-4/20. Finally, to illustrate the use of MeioSeed, salt stress was investigated as a novel trigger for changes in crossover frequency, through which we found that salt stress can increase crossover frequency between the fluorescence marker genes in Col3-4/20, and can induce genome-wide fluctuations in crossover frequency.

The combined use of CellProfiler and an Ilastik-based classifier by MeioSeed is fairly new, and has only to a small extent been explored for image analysis [24]. Previously, seed counting and image analysis relied largely on the use of other software packages such as ImageJ [32, 33], or the combined use of Adobe Photoshop and ImageJ [32]. CellProfiler has previously been coined as the best program to reliably count seeds of various crop species [34], especially because it has a higher accuracy and can be used on images of different sources whereas ImageJ is most suitable for digital camera images [34]. In addition, establishing an automated and high throughput seed counting and classification algorithm in ImageJ requires quite extensive programming experience, whereas CellProfiler allows for user friendly, module-based and linear image analysis pipelines which can easily be fine-tuned and optimized without programming knowledge. As presented here, the use of CellProfiler can also be integrated with Ilastik, allowing for very robust pixel classification and therefore more efficient recognition of seeds. It has to be noted, however, that CellProfiler lacks scripting possibilities to enable more complex workflows (e.g. involving programming loops). When combined with a robust seed classifier, CellProfiler therefore likely allows for the best available recognition and counting of seeds. A disadvantage of CellProfiler, however, is that it can be relatively slow at counting large image files. In order to have MeioSeed fluently running, we experienced that this at least requires a quadcore processor.

In the present study we have described and optimized the settings of MeioSeed for images generated by the LeicaMZ16FA fluorescence stereomicroscope. In principle, MeioSeed is compatible with images generated by any type of fluorescence microscope or digital camera setup, given that the exposure settings are such that seeds are clearly recognizable from the images. In case MeioSeed is unable to count seeds from images generated by another microscope, it is likely that optimization of the exposure settings is required. Here, we have optimized the ‘FilterObjects’ fluorescence thresholding settings (Fig. 3) based on a rather specific set of exposure settings for the LeicaMZ16FA, so it is likely that these thresholds are very different when another microscope with other exposure settings is used. We have noticed that the classifier is robust enough to deal with substantial deviations in the protocol. However, even though the classifier is very robust, it is likely that another microscope setup requires reoptimization of the ‘FilterObjects’ settings to accurately recognize the seeds and for the output to match the theoretical segregation pattern of the transgenes. Furthermore, in our experience Col3-4/20 seeds and Col3-4/20 × Ler-0 hybrids seeds display hardly any or even no autofluorescence at all in both the RFP and the GFP channels, but this might of course be different for other reporter lines or in other hybrid backgrounds. In such cases counting seeds requires assessment of autofluorescence levels of the seeds and optimization of the ‘FilterObjects’ settings to match the relevant model for recombination frequency and transgene segregation. With higher levels of autofluorescence finding a good fit between the ‘FilterObjects’ settings and the model might require more fine-tuning.

The seed classifier of MeioSeed was trained by machine-learning to specifically recognize the morphology and shape of *Arabidopsis* seeds, and to exclude other objects from the data analysis. This was done in an iterative process of learning from manually labelled ‘White’ images of seeds. Although the final batch processing of MeioSeed was for our purpose based on recognition of *Arabidopsis* seeds through the classifier, it can in principle be adapted to function for the seeds of any other plant species or for any other type of object (e.g. pollen, vegetative tissue, callus). This would in principle only require training of a classifier in Ilastik with ‘White’ images of those seeds or objects, and subsequently optimizing the ‘FilterObjects’ settings for accurate recognition depending on the levels of fluorescence reporter gene expression and background fluorescence. In fact, the classifier file for *Arabidopsis* seeds which was generated in this study can already be used for seeds of other plant species or for counting any other objects, but the performance of MeioSeed will drop along with increasing differences in shape and size. It might be very worthwhile to consider the combined use of CellProfiler and Ilastik for seed counting purposes in crop species, as we have shown in the present study for rapeseeds and barley grains (Additional files 3 and 4). Seed count is considered to be an important agronomic parameter and is commonly used as a measure of yield [34, 35]. For applications in meiosis research, similar to our present study, there are to our knowledge no seed reporter lines harboring *in cis* linked fluorescence reporter genes yet available for crop species. Such lines might very well become available for any given crop species, but it might be technically more challenging than for the model plant *Arabidopsis*. Obviously, the efficiency of fluorescence detection is likely to depend on the thickness of the seed coat and on the amount of endosperm relative to the size of the embryo; thin-walled A*rabidopsis* seeds which also contain very little endosperm thus allow for a clear view on the embryos representing the F_2_ population that express the fluorescence markers. For other species, the situation is likely to be less favorable.

Using MeioSeed we have found that salt stress can act as a novel abiotic modulator of crossover frequency in *Arabidopsis*. Our data therefore indicate that fluorescent seed or pollen reporter lines are viable options to screen for novel abiotic treatments to enhance crossover frequency in plants. In principle this just requires the exposure of F_1_ plants (homozygous reporter line × other homozygous accession) to abiotic treatments and fluorescence microscopy analysis of the F_2_ seed pool or the F_1_ pollen. It has to be noted, however, that screening for treatments using fluorescence reporter lines will require further investigation of candidate F_2_ populations by genotyping and/or sequencing to get a complete overview of the genome-wide impact of the treatment, and of the actual positions and the number of crossovers. In addition, whether or not an effect on crossover frequency can be observed in fluorescence reporter lines might depend on the position of the markers in the genome, meaning that it might be worthwhile to screen for treatments using multiple reporter lines in parallel. Previously, temperature stress was reported to enhance crossover frequency in *Arabidopsis* using a fluorescent pollen reporter line [17] and fluorescent seed reporter lines [36], further indicating that other abiotic stresses (e.g. light stress, drought stress and osmotic stress) could also be explored as novel modulators of crossover frequency. This is supported by the fact that abiotic stresses are triggers of chromatin remodeling in plants [29, 30], which is important for the accessibility of a genomic locus to a crossover event [37]. From a more evolutionary and ecological point of view, one could also hypothesize that it would be beneficial for a plant to upregulate the frequency of crossovers upon experiencing abiotic stress to genetically diversify its offspring. Evidence for a similar hypothesis has recently been found in the case of biotic stress, where recombination rate was found to be enhanced at disease resistance loci [38].

## Conclusions

We have shown that MeioSeed allows for the high throughput counting of green and red fluorescent seeds for meiotic crossover analysis, and presented an overview of the optimization steps for our microscopy setup. These settings could be adjusted and optimized for any other fluorescence microscopy setup. In addition, we showed that MeioSeed can also be used for other plant species by retraining the classifier for those seeds, and can thus in principle be used for crossover frequency analysis in other types of seeds as well, provided that reporter lines become available. Using salt stress as an example, we showed that MeioSeed in combination with fluorescence reporter lines can be used to screen for novel treatments affecting crossover frequency in *Arabidopsis*.

## Additional files

**Additional file 1.** Operation and processing steps of the MeioSeed Algorithm. Additional text from the results section describing how MeioSeed should be operated and which steps the CellProfiler algorithm takes to process fluorescence microscopy images into images with seed counts.

**Additional file 2.** Genetic maps of F_2_ populations resulting from treatment of Col3-4/20 (♂) × Ler-0 (♀) F_1_ hybrids with either 0 mM or 300 mM NaCl, generated by KASP genotyping with 39 SNP marker sets (384 chromosomes per treatment).

**Additional file 3.** Overview of rapeseeds (*Brassica napus* cv. Westar) counted with MeioSeed. The same settings as for *Arabidopsis* seeds were used, except for a seed classifier file which was trained in Ilastik to recognize rapeseeds.

**Additional file 4.** Overview of barley grains (*Hordeum vulgare*) counted with MeioSeed. The same settings as for *Arabidopsis* seeds were used, except for a seed classifier for a seed classifier file which was trained in Ilastik to recognize barley grains.
