# Supplementary material for: MeioSeed: a CellProfiler-based program to count fluorescent seeds for crossover frequency analysis in Arabidopsis thaliana
Source: Plant Methods. 2018 Apr 18;14:32. doi: 10.1186/s13007-018-0298-3 (PMC5905130; doi:10.1186/s13007-018-0298-3)
Supplement: Supplementary file 1 — Additional file 1. Operation and processing steps of the MeioSeed Algorithm. Additional text from the results section describing how MeioSeed should be operated and which steps the CellProfiler algorithm takes to process fluorescence microscopy images into images with seed counts. [file 13007_2018_298_MOESM1_ESM.pdf]

Additional File 1

Operation and processing steps of the MeioSeed algorithm

MeioSeed is started by opening the CellProfiler file and clicking ‘View output settings’, where the input folder holding the TIFF microscopy images and an output folder for the generated data can be selected. The ‘White’, ‘Red’ and ‘Green’ fluorescence microscopy images (Fig.2, step 1) are imported by CellProfiler from the designated input folder. It is important to note that the file names of the ‘White’, ‘Red’ and ‘Green’ images of a given sample should be in the format 0001-White, 0001-Red and 0001-Green for correct recognition as a set. Subsequently, the classifier file should be pasted in a folder which can be designated in the ‘Classifier file name’ line at processing step ‘ClassifyPixels’. Finally, the thresholds for red and green fluorescence can be set at the processing steps ‘FilterObjects’ in lines ‘Minimum value’. Optimization of these settings is discussed further below. The program can now be run by clicking on ‘Analyze images’.

The RGB microscopy images are subsequently converted to 8-bit gray images and pixel values are rescaled to the maximal value in the pixel intensity histogram to reduce overall differences in fluorescence intensity between seeds (Fig. 2, step 2). In this way, we could ensure that there is no bias for the recognition of strongly fluorescent seeds in subsequent processing steps. The rescaled ‘White’ image is used to create masks for individual seeds in the rescaled ‘Red’ and ‘Green’ images. Based on the Ilastik classifier, CellProfiler then generates probability maps for the pixels in the images, displaying the likelihood of each pixel being part of the ‘Seed’ class, the ‘SeedEdge’ class or the ‘Background’ class, with black representing a probability of 0% and white a probability of 100%. (Fig. 2, step 3). The class ‘SeedEdge’ was introduced to improve the recognition of directly adjacent seeds. The probability map image of the ‘Seed’ class is segmented by a manual threshold of 0.5 (probability 50%) to create a binary image and subsequently cleaned with image filters taking into account seed morphology. The probability maps are then thresholded for object size to eliminate non-seed objects from the analysis, resulting in images with primary seed objects only (Fig. 2, step 4).

The primary seed objects are used to create more accurate seed objects by propagation in the binary inverted ‘Background’ images (manual threshold of background probability map image was set at 0.5) (Fig. 2, step 5) and are eroded by five pixels to prevent the edges of other seeds from being included in the final area per seed. To tune the sensitivity and specificity of the pipeline for counting ‘RedOnly’,

‘GreenOnly’ and ‘Red and Green’ seeds, a seed object filter step called ‘FilterObjects’ was introduced to set a threshold for red and green fluorescence intensity (Fig. 2, step 6). The optimization of the ‘FilterObjects’ settings for Arabidopsis seeds is described in the main text. MeioSeed generates .tiff images in the designated output folder in which the seed counts and fluorescence signals are displayed as overlays on the original ‘White’ microscopy images (Fig. 2, step 7).
