# Supplementary material for: MeioSeed: a CellProfiler-based program to count fluorescent seeds for crossover frequency analysis in Arabidopsis thaliana
Source: Plant Methods. 2018 Apr 18;14:32. doi: 10.1186/s13007-018-0298-3 (PMC5905130; doi:10.1186/s13007-018-0298-3)
Supplement: Supplementary file 2 — Additional file 2. Genetic maps of F2 populations resulting from treatment of Col3-4/20 (♂) × Ler-0 (♀) F1 hybrids with either 0 mM or 300 mM NaCl, generated by KASP genotyping with 39 SNP marker sets (384 chromosomes per treatment). [file 13007_2018_298_MOESM2_ESM.pdf]

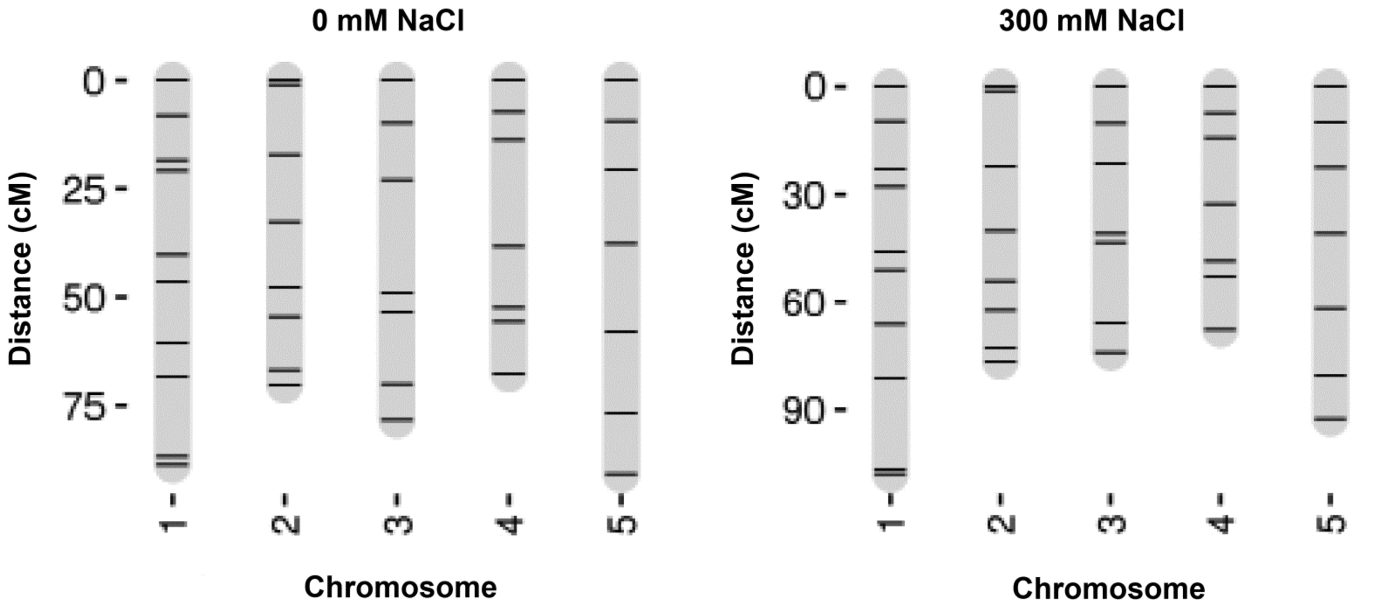

Additional File 2. Genetic maps of F2 populations resulting from treatment of Col3-4/20 () x Ler-0 () F_1 hybrids with either 0 mM or 300 mM NaCl, generated by KASP genotyping with 39 SNP marker sets (384 chromosomes per treatment).
