# Supplementary material for: MeioSeed: a CellProfiler-based program to count fluorescent seeds for crossover frequency analysis in Arabidopsis thaliana
Source: Plant Methods. 2018 Apr 18;14:32. doi: 10.1186/s13007-018-0298-3 (PMC5905130; doi:10.1186/s13007-018-0298-3)
Supplement: Supplementary file 3 — Additional file 3. Overview of rapeseeds (Brassica napus cv. Westar) counted with MeioSeed. The same settings as for Arabidopsis seeds were used, except for a seed classifier file which was trained in Ilastik to recognize rapeseeds. [file 13007_2018_298_MOESM3_ESM.pdf]

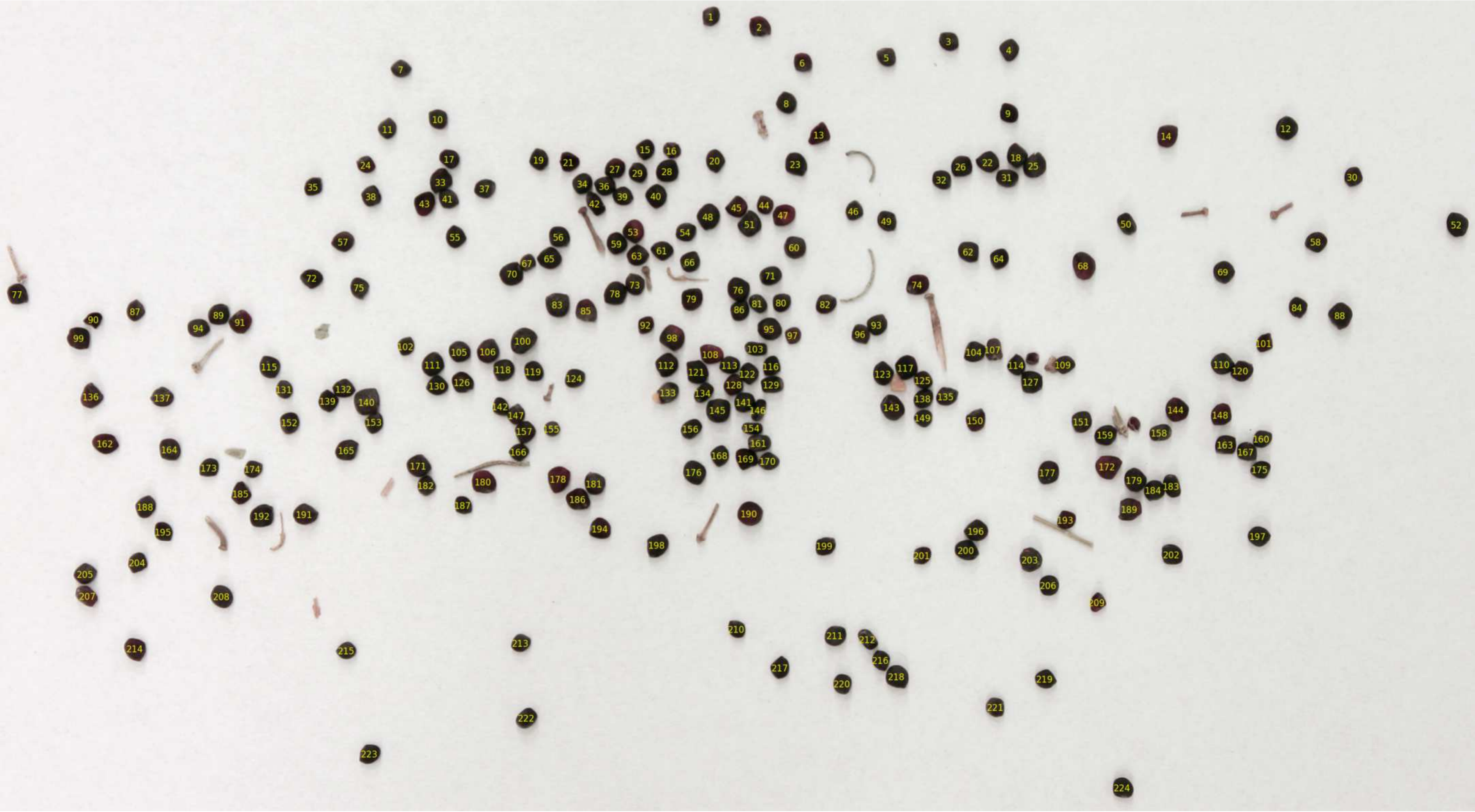

Additional file 3. Overview of rapeseeds (*Brassica napus* cv. Westar) counted with MeioSeed. The same settings as for Arabidopsis seeds were used, except for a seed classifier file which was trained in Ilastik to recognize rapeseeds.
